# On the Destruction of the Dental Pulp by the Heat of Electricity

**Published:** 1851-10

**Authors:** Thomas H. Harding


### ARTICLE XIV.

### *On the Destruction of the Dental Pulp by the Heat of Electricity.* By Thomas H. Harding, Esq.

It may be confidently stated, that a more rapid, certain, and safe method of destroying the sensitive pulp of a decayed tooth, than any with which dentists are already familiar, will be regarded as a great advantage by all engaged in the practice of dental surgery. Having read in your journal an abstract of a paper communicated to the Medico-Chirurgical Society by Mr. Marshall, giving an account of his method of employing the heat of electricity for the purpose of limited cauterization in surgical disease, and a subsequent report of several operations performed by him, it struck me that a platina wire, heated in the way he recommends, might be made available for the instantaneous destruction of an exposed tooth-pulp.

On communicating this idea to Mr. Marshall, I found that it had already occurred to himself, and had been mentioned by him as one obvious application of his method of operating with the electric heat. Moreover, his experience in the matter enabled him to suggest for the purpose a very simple and suitable apparatus, which is, in fact, a reduced copy of that which he had used so successfully in other cases, and in which the cauterizing portion consists of a flattened *loop* of fine platina wire.

The apparatus as ultimately employed by me may be thus described:—The battery, which of course may vary according to the choice of the operator, but which it is so desirable to render as elegant and simple in arrangement as possible, is constructed on a plan similar to that of the larger battery now employed by Mr. Marshall, which I believe he shortly intends to describe. It consists of only two pairs of plates, contained in a single cell, and is set in action by one fluid—viz. dilute sulphuric acid. The terminal six inches of the poles, which are of copper wire, plated, are supported on an ebony or ivory handle, upon the side of which one of the poles is interrupted at a particular point. The extremities of the poles are connected by a piece of platina wire, one-hundredth of an inch thick and three-quarters of an inch long, which is bent into a *loop*. The sides of this loop are then brought parallel and nearly close to each other without touching, and it is thus introduced into the pulp cavity of the tooth to be operated on. By a slight pressure on one side of the handle, the interrupted pole is temporarily joined, and the platina wire immediately becomes brilliantly heated as it lies in contact with the tooth-pulp. Sometimes, however, I have found it desirable, in the first place, to complete the galvanic circuit, and thus heat the platina wire, before bringing it to bear upon the exposed pulp. The flexibility of the loop of wire enables the operator to bend it in any direction previously to use. In this way I have succeeded in rapidly destroying the pulps of decayed and condemned teeth and have proceeded, after a few minutes, to the operation of filling with gold or with Ash's metallic paste. I have also destroyed, with the greatest ease and rapidity, the pulps of incisor teeth, cut off for the purpose of being pivoted.

It is obvious that this method is applicable either for the simple cure of tooth-ache, or as a preliminary step to the operation of filling. I am aware that there is nothing novel in the use of a hot wire for destroying the nerve of an aching tooth. The old village doctoress has long ago cured tooth-ache by the thrust of a hot needle or pin, and dentists occasionally use a heated wire. But the difficulty has been felt of applying the wire easily, and at a duly elevated temperature. In the method just described, this is surely, readily, and instantaneously accomplished. The vitality of the tooth-pulp is thoroughly destroyed, and it is even so far consumed or carbonized that the operations of filling or pivoting may be at once proceeded with, instead of having to be delayed. Owing to the extreme fineness of the wire employed, the local heat, though intense, is very limited in its action, and with due care the tooth substance need not suffer any appreciable injury. It may also be remarked, that in operating on some teeth, I have found, by completing the galvanic circuit on approaching the tooth, that the light given out by the incandescent wire aids very remarkably in giving a perfect view of the exact point at which the tooth-pulp is exposed.

In conclusion, I can only say, I beg confidently to lay this method before the notice of the profession, as the most ingenious and simple contrivance yet invented for the object in view, and as one which I think will be very generally adopted. It will give me great pleasure to exhibit the apparatus and the mode of operating to any who are interested about it; and I may add that the apparatus itself will be constructed by Mr. Coxeter, of Grafton-street East.

